# Radiological Appearance and Imaging Techniques in the Diagnosis of Advanced Central Pontine Myelinolysis

**DOI:** 10.7759/cureus.30328

**Published:** 2022-10-15

**Authors:** Cleofina Furtado, Sanjeev Nayak, Changrez Jadun, Sachin Srivastava, Zafar Hashim

**Affiliations:** 1 Department of Diagnostic and Interventional Radiology, University Hospitals of North Midlands NHS Trust, Stoke On Trent, GBR

**Keywords:** magnetic resonance imaging (mri), computed tomography (ct), hyponatremia, extra pontine myelinolysis (epm), central pontine myelinolysis (cpm)

## Abstract

Conventional magnetic resonance imaging (MRI) and computed tomography (CT) are used to diagnose central pontine myelinolysis (CPM), which is seen in the setting of osmotic changes, typically with the rapid correction of hyponatremia. However, they typically follow clinical symptoms and fail to detect myelinolytic lesions within the first two weeks, limiting their efficacy in early diagnosis. CPM can mimic brainstem ischaemic changes on CT head and a glioma on MRI. This case reviews the relationship between radiological changes seen with clinical symptoms and serum sodium levels, combined with reviewing pioneering advances in radiomic analysis, including diffusion-weighted MRI, CT brain perfusion and MR spectroscopy.

## Introduction

Central pontine myelinolysis (CPM) is a rare clinical syndrome characterised by the pontine white matter tract and extra pontine myelinolysis (EPM) with a variety of clinical manifestations [1]. CPM has been reported in association with electrolyte abnormalities as well as in the context of liver transplantation, lithium poisoning and carbamate poisoning [2-4]. Patients who are alcoholic or malnourished are usually lacking in organic osmolytes, which puts them at a higher risk of developing osmotic demyelination syndrome [2]. The most common clinical setting for CPM is the rapid correction of severe hyponatraemia when serum sodium in patients with chronic hyponatremia is rapidly rectified, resulting in the rapid rise of plasma osmolality [5]. Patients with adrenal insufficiency, metabolic disorders, malnutrition and cancer are also thought to be particularly susceptible to this condition [6].

Clinical outcomes in CPM might range from a moderate motor impairment that resolves entirely over time to severe locked-in syndrome [7]. Quadriparesis is caused by the involvement of the corticospinal pathways, which is initially flaccid but eventually becomes spastic [8]. Pseudobulbar palsy caused by corticobulbar tract involvement causes head and neck paralysis as well as dysphagia and dysarthria [9]. Lesions in the pontine tegmentum or thalamus produce come and disorientation, as well as a locked-in condition, marked by paralysis of the lower cranial nerves and limb muscles [7].

We described a patient whose treatment for severe hyponatremia was complicated by clinical and radiological signs of CPM.

## Case presentation

A 37-year-old, otherwise healthy, male with a history of chronic alcoholism (30-40 units daily) presented with a fall and confusion.

Blood tests demonstrated hypokalaemia (K - 2.6 mmol/L), hyponatremia (Na - 113 mmol/L), acute kidney injury (estimated glomerular filtration rate (eGFR) 56 mL/min/1.73m*2 and creatinine 138 umol/L), low vitamin B12 and magnesium (B12 - 913 pg/mL, Mg - 0.62 mmol/L) but normal folate levels. He was treated with IV 0.9% normal saline with KCl. The CT head was unremarkable on this admission. Eventually, sodium improved with an upward trend of 6-7 mmol/L per day and baseline after five days of therapy. The potassium, magnesium and renal functions recovered to normal, and the patient was discharged home.

He presented again five weeks later with bilateral lower limb weakness and unsteadiness. The biochemical profile, including sodium, was within normal limits.

**Figure 1:**
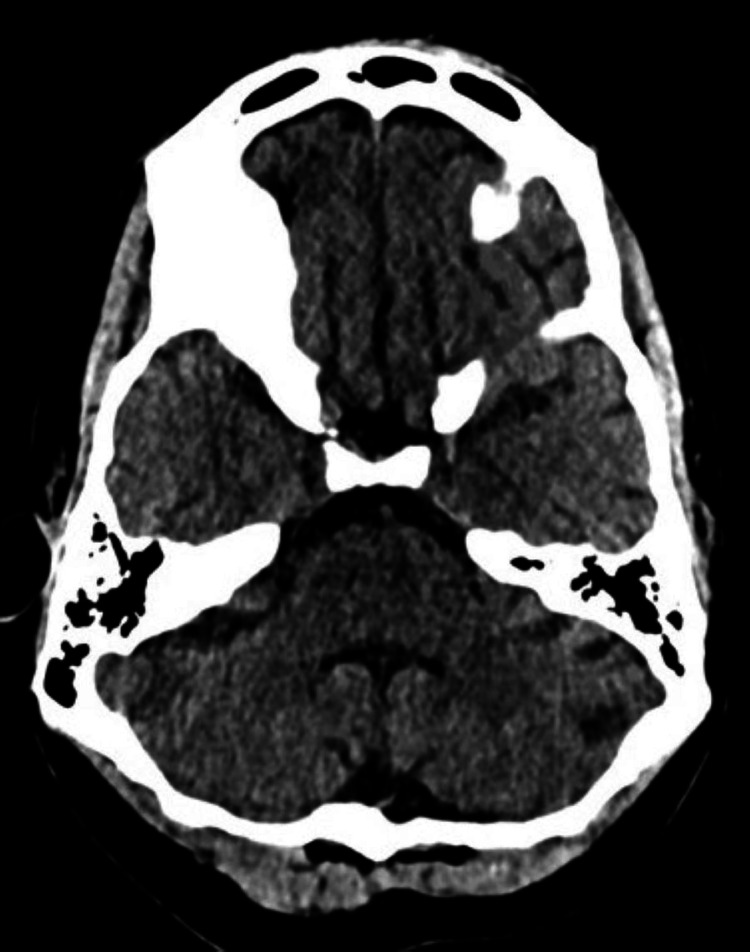
Axial plain CT head at initial admission was reported as nil acute with no evidence of any abnormality

**Figure 2:**
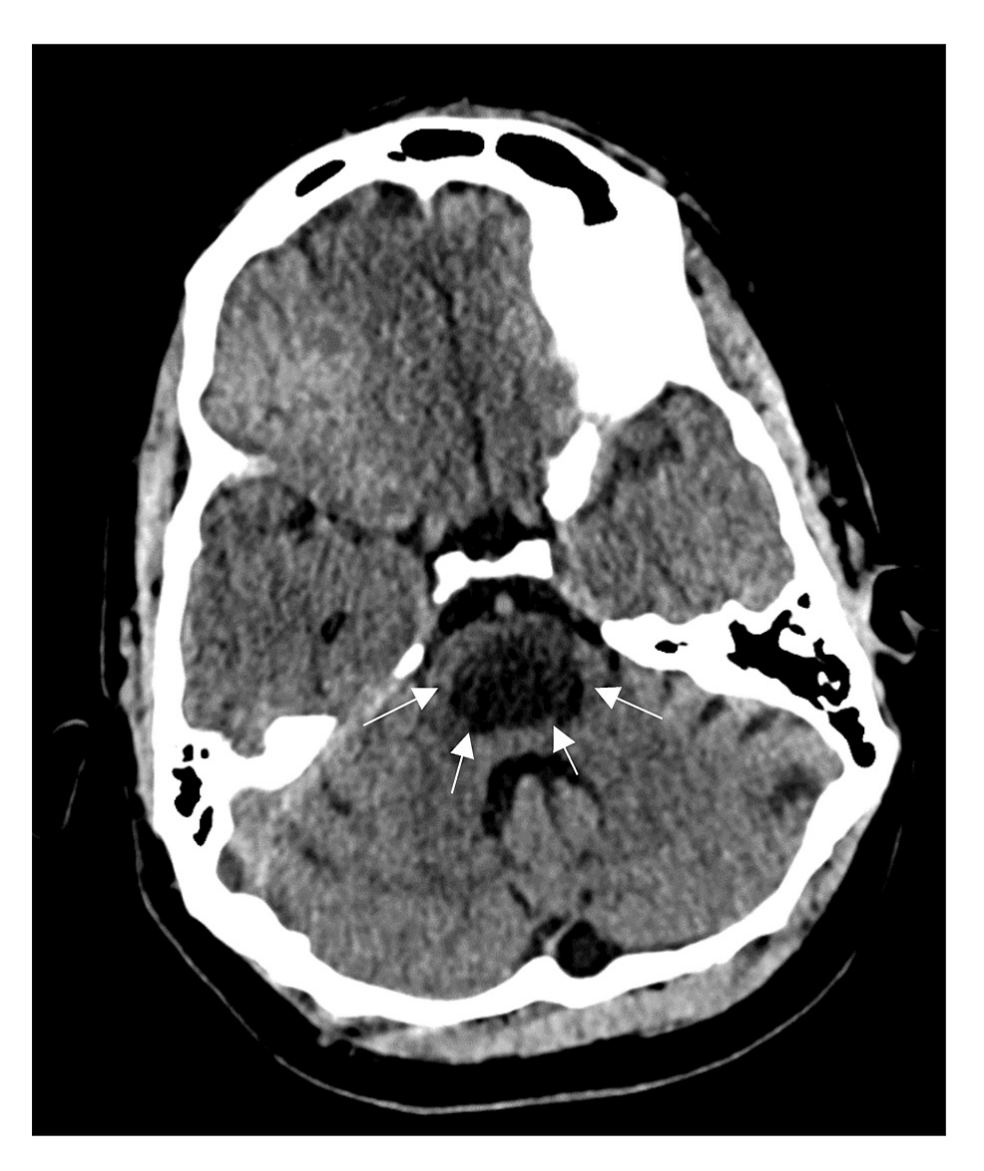
Axial plain CT head at the second admission 5 weeks later demonstrates central low attenuation within the lower pons (between white arrows)

**Figure 3:**
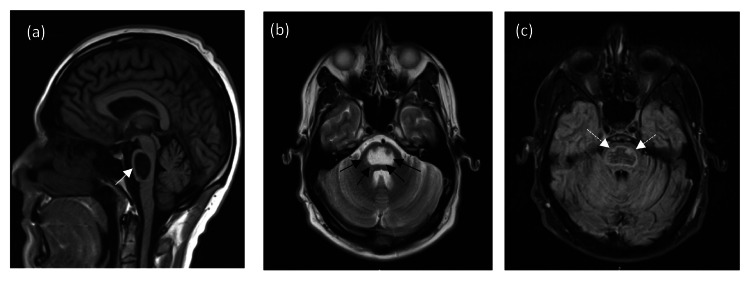
MRI brain: sagittal T1 (a) demonstrates central pons low signal intensity (white arrow). Corresponding axial T2 (b) shows high signal (between black arrows) and trident-shaped FLAIR (c) high signal intensity (dashed white arrows) surrounding the central low signal intensity in keeping with encephalomalacia changes. FLAIR: fluid-attenuated inversion recovery

**Figure 4:**
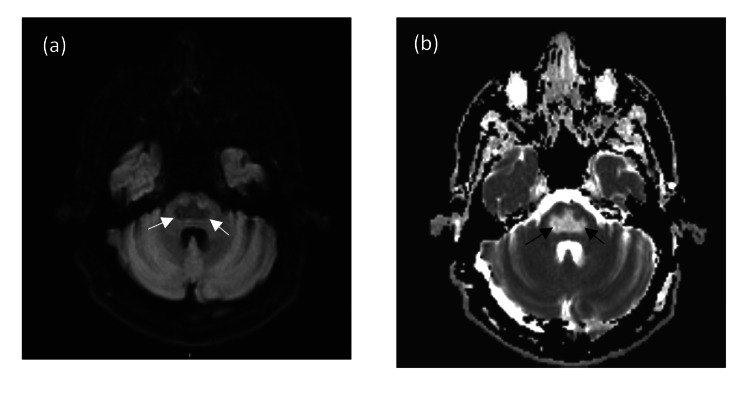
MRI brain: axial DWI (a) and ADC (b) demonstrates T2 shine through with low signal on DWI (between white arrows) and corresponding high signal on ADC map (between black arrows) DWI: diffusion-weighted imaging; ADC: apparent diffusion coefficient

CT brain demonstrated low attenuation crossing the midline in the lower pons (Figure 1). There were no signs of oedema or worsening intracranial pressure. Although there was faint effacement of the left lateral fourth ventricle, there were no features of hydrocephalus. The MRI brain showed a classic central symmetrical trident shape with a high signal on T2-weighted imaging (T2WI) and a low signal on fluid-attenuated inversion recovery (FLAIR) imaging sequence (Figure 2). On T1-weighted imaging (T1WI), there was corresponding central low signal intensity. The descending corticospinal tracts were preserved. The diffusion-weighted imaging (DWI) sequence demonstrated only faint restricted diffusion outlining the lesion depicting the well-preserved descending corticospinal tracts (Figure 3). Thus, a diagnosis of central pontine myelinolysis was made. Figure 4 shows DWI and apparent diffusion coefficient (ADC) mapping.

His neurological function improved over time, and he was discharged 10 days following admission. He had almost complete functional recovery and was independent in all activities of daily living.

## Discussion

The fundamental pathology identified in CPM is compression and subsequent demyelination of fibre tracts due to either a diminished adaptation capacity of the neuroglia to substantial shifts in serum osmolarity or cellular oedema produced by changes in electrolyte gradients [10,11]. A dense grid-like arrangement of white and grey fibres in the central pontine region makes it particularly susceptible to osmotic demyelination [8]. Compression of fibre tracts caused by cellular oedema produced by shifting osmotic pressures may result in demyelination [12]. EPM occurs in the areas of greatest grey and white admixture and is seen involving the midbrain, thalamus, basal nuclei and cerebellum [7,8].

Conventional MRI (T1WI, T2WI and FLAIR) and CT are used to diagnose CPM, however, they typically follow clinical symptoms and fail to detect myelinolytic lesions within the first two weeks, limiting their efficacy in early CPM diagnosis [13]. Furthermore, CT imaging is far less sensitive than MRI imaging for detecting early CPM abnormalities, where the diagnosis of CPM cannot be ruled out in the presence of normal CT imaging, and hence, if high clinical suspicion further imaging should be recommended [14].

DWI, on the other hand, can identify anomalous diffusion restriction in demyelination lesions within 24 hours after initiation, which can boost diagnostic confidence [15]. In severe cases, however, the pontine lesions may display diffusion restriction, which is caused by cell necrosis and might be mistaken for pontine infarct [14].

Low T1WI signal intensity and high T2WI FLAIR sequence with an oval- or trident-shaped (trident sign) or the snout of a pig (piglet sign) are characteristic of CPM [16]. This is due to the relative sparing of the descending corticospinal and corticobulbar tracts [16]. In EPM, T2WI and FLAIR sequences show symmetrical high signal abnormalities in the bilateral caudate nucleus and putamen with sparing of the globus pallidus [15]. Additionally, there is no contrast enhancement after intravenous gadolinium administration in both CPM and EPM [1].

CT perfusion demonstrates increased blood flow, decreased mean transit time and decreased time to the maximum in the pons, which might be attributed to higher metabolic demands at the location of cell injury [16]. This in addition to microglia inflammation shows avid fluorodeoxyglucose (FDG) absorption in the pons on a PET scan performed at two weeks [17]. On magnetic resonance spectroscopy, the acute phase is marked by a drop in the N-acetyl aspartate (NAA)/creatine (Cr) ratio, an increase in the choline (Cho)/Cr ratio and a lactate/lipid peak. This displays a further rise in the Cho/Cr ratio and a decrease in the NAA/Cr ratio in the later phase [18].

Pontine lesions discovered incidentally on MRI scans are a diverse group, with many of them more consistent with pontine ischemia rarefaction than with asymptomatic CPM [19]. With the right clinical information, this result can be used to make an early CPM diagnosis [19]. The imaging features could also mimic a glioma due to similarity in the pathological aetiology resulting from damage in the blood-brain barrier, which produces abnormal inhomogeneous signal intensity on contrast-enhanced MRI. In such cases, follow-up could be beneficial for a definite diagnosis [18].

## Conclusions

There is a high index of suspicion for CPM and a low threshold for MRI in symptomatic patients with current or recent hyponatremia regardless of the appropriate correction rate. CPM imaging appearances may be delayed in some patients and if clinical suspicion is high, repeat imaging in 10-14 days is recommended. The imaging appearance of CPM can mimic brainstem ischaemic changes on CT head and mimic a glioma on MRI. In patients with a background of hyponatremia, CPM should be considered. In advanced CPM, MRI may demonstrate appearances of encephalomalacia with a T2 shine-through rather than characteristic diffusion restriction.
